# Lipoxins as biomarkers of lupus and other inflammatory conditions

**DOI:** 10.1186/1476-511X-10-76

**Published:** 2011-05-15

**Authors:** Undurti N Das

**Affiliations:** 1 UND Life Sciences, 13800 Fairhill Road, #321, Shaker Heights, OH 44120, USA; 2 School of Biotechnology, Jawaharlal Nehru Technological University, Kakinada-533 003, India; 3 Krishna Institute of Medical Sciences, Secunderabad-500 003, India; 4 Bio-Science Research Centre, GVP College of Engineering Campus, Madhurawada, Visakhapatnam-530 048, India

**Keywords:** Lupus, rheumatoid arthritis, diabetes mellitus, multiple sclerosis, lipoxins, resolvins, protectins, maresins, autoimmunity, cytokines, tumor necrosis factor, free radicals, prostaglandins

## Abstract

Inflammatory events persist in systemic lupus erythematosus (lupus) despite the use of anti-inflammatory (both steroidal and non-steroidal) and immunosuppressive drugs leading to delay in the healing/repair process and so tissue/organ damage continues. The continuation of inflammation in lupus could be attributed to failure of the resolution process due to deficiency of potent endogenous pro-resolution-inducing molecules such as lipoxin A_4 _(LXA_4_). It is likely that progression and flares of lupus and lupus nephritis are due to decreased formation and release of LXA_4_. Hence, administration of LXA_4 _and its analogues could be of benefit in lupus. Furthermore, plasma and urinary measurement of lipoxins may be used to predict prognosis and response to therapy. It is likely that lipoxins and other bioactive anti-inflammatory lipids such as resolvins, protectins, maresins and nitrolipids play a significant role in other auto-immune diseases such as rheumatoid arthritis, type 1 diabetes mellitus and multiple sclerosis and hence, could be of significant benefit in these diseases.

## Introduction

Systemic lupus erythematosus (SLE), a disease of unknown aetiology that is more common in women than in men, is characterized by non-destructive arthritis/arthralgias, a cutaneous rash, vasculitis, involvement of the central nervous system (CNS) and renal and cardiopulmonary manifestations. Although genetic, environmental and sex hormonal factors have been implicated in the pathogenesis of SLE (also called as "lupus"), it is known that several cytokines, nitric oxide (NO), free radicals, a deranged immune system, a deficient anti-oxidant defenses, and Toll-like receptors have a significant role both in the initiation and perpetuation of the inflammatory process observed.

The fundamental process in lupus appears to be rendering DNA and RNA antigenic that leads to the production of anti-DNA and anti-RNA antibodies and the formation of immune complexes. These antibodies and immune complexes, in turn, trigger both a local and systemic inflammatory response that ultimately leads to target organ/tissue damage seen in lupus. The susceptibility to develop lupus in a given individual seems to have, at least, partly a genetic basis though this is still not very clear. Once the inflammatory process is triggered, this leads to the production of a variety of pro-inflammatory cytokines such as interleukin-1 (IL-1), IL-6, tumor necrosis factor-α (TNF-α), interferons (IFNs), macrophage migration inhibitory factor (MIF), HMGB1 (high mobility group B1) and possibly, a reduction in the elaboration of anti-inflammatory cytokines such as IL-10, IL-4, and transforming growth factor-β (TGF-β). This imbalance between the pro- and anti-inflammatory cytokines coupled with increased secretion of free radicals such as superoxide anion (O_2_^-^.), hydrogen peroxide (H_2_O_2_), singlet oxygen, inducible nitric oxide (iNO), and other reactive oxygen species (ROS) by activated monocytes, macrophages, polymorphonuclear leukocytes (PMNL), T cells, Kupffer cells, glial cells in the brain, and other organ specific reticuloendothelial cells would ultimately cause target tissue/organ damage seen in lupus.

I propose that continued inflammatory events seen in lupus could be due to failure of the resolution of inflammation. Thus, the balance between inflammation and resolution is disturbed more in favor of pro-inflammatory events and/or failure of resolution inducing molecules to be produced at the most appropriate time leading to non-resolution of inflammation. In other words, even after the inciting agent responsible for the initiation of inflammation is removed; inappropriate inflammation continues simply because resolution failed to occur. This leads to delay in the healing/repair process and so tissue/organ damage continues. This may explain as to why even when these patients are continuing to take anti-inflammatory and immunosuppressive medicines, target organ damage continues. In view of this, it is imperative that institution of pro-resolution-inducing agents needs to be employed to obtain full remission and restore normal physiological function of the target tissues/organs in these diseases. Such endogenous pro-resolution-inducing molecules include: lipoxins, resolvins, protectins, maresins, nitric oxide, nitrolipids, 15 deoxyΔ^12-14 ^PGJ_2_, PGD_2_, anti-inflammatory cytokines such as IL-4, IL-10, and some polyunsaturated fatty acids (PUFAs).

Based on this hypothesis, it is suggested that progression and flares of lupus are due to increased production of pro-inflammatory molecules IL-6, TNF-α, MIF (macrophage migration inhibitory factor), HMGB1 (high mobility group box 1), free radicals and lipid mediators such as prostaglandins (PGs), leukotrienes (LTs) and/or decreased formation and release of anti-inflammatory molecules: IL-4, IL-10, TGF-β, and lipoxins, resolvins, protectins, maresins and nitrolipids.

### Metabolism of essential fatty acids with specific reference to inflammation

Cis-Linoleic acid (LA, 18:2 ω-6) and α-linolenic acid (ALA, 18:3 ω-3) are essential nutrients since they cannot be synthesized by the human body and hence are called as "essential fatty acids" (EFAs). LA is converted to gamma-linolenic acid (GLA, 18:3, ω-6) by the action of the enzyme Δ^6 ^desaturase, and GLA is elongated to form di-homo-GLA (DGLA, 20:3, ω-6), the precursor of the 1 series of prostaglandins. Δ^6 ^desaturase is the rate-limiting step in the metabolism of EFAs. DGLA can also be converted to arachidonic acid (AA, 20:4, ω-6)) by the action of the enzyme Δ^5 ^desaturase. AA forms the precursor of 2 series of prostaglandins, thromboxanes and the 4 series leukotrienes (LTs). ALA is converted to eicosapentaenoic acid (EPA, 20:5, ω-3) by Δ^6 ^and Δ^5 ^desaturases. EPA forms the precursor of the 3 series of prostaglandins and the 5 series of LTs. EPA can be elongated to form docosahexaenoic acid (DHA, 22:6, ω-3). AA, EPA and DHA also form precursors to a group of novel compounds: lipoxins, resolvins, protectins and maresins [1-9] that have anti-inflammatory action. Eicosanoids bind to G protein-coupled receptors on many cell types and mediate virtually every step of inflammation, are found in inflammatory exudates, and their synthesis is increased at sites of inflammation. Non-steroidal anti-inflammatory drugs (NSAIDs) such as aspirin inhibit cyclo-oxygenase (COX) activity and thus, are believed to bring about their anti-inflammatory action.

### Lipoxins, resolvins, protectins and maresins

There are two cyclo-oxygenase enzymes, the constitutively expressed COX-1 and the inducible enzyme COX-2. Different types of PGs are formed by the action of COX enzymes depending on the substrate fatty acid from which they are derived. Different types of PGs have different actions and sometimes diametrically opposite actions. For example, PGE_2_, PGF_2α_, thromboxane A_2 _(TXA_2_), and leukotrienes (LTs) have pro-inflammatory actions whereas PGE_1 _and prostacyclin (PGI_2_) show anti-inflammatory actions. Furthermore, the distributions of COX-1 and COX-2 enzymes have restricted tissue distribution. Platelets contain thromboxane synthetase, and hence TXA_2_, a potent platelet-aggregator and vasoconstrictor, is formed in these cells, whereas vascular endothelial cells possess PGI_2 _synthetase but lack thromboxane synthetase and thus, they form, mainly, PGI_2_, a potent platelet anti-aggregator and vasodilator. The balance between TXA_2 _and PGI_2 _is important in thrombus formation in coronary and cerebral blood vessels. PGD_2_, PGE_2 _and PGF_2α_, major metabolites of the COX pathway have pro-inflammatory actions.

There are 3 types of lipoxygenases and are present in only a few types of cells. 5-lipoxygenase (5-LO), present in neutrophils, produces 5-HETE, which is chemotactic for neutrophils, and is converted into leukotrienes (LTs). LTB_4_, a potent chemotactic and activator of neutrophils, induces aggregation and adhesion of leukocytes to vascular endothelium, generation of ROS, and release of lysosomal enzymes. The cysteinyl-containing leukotrienes C_4_, D_4_, and E_4 _(LTC_4_, LTD_4_, and LTE_4_) induce vasoconstriction, bronchospasm, and vascular permeability in venules. LTs are more potent than histamine in increasing vascular permeability and causing bronchospasm. LTs mediate their actions by binding to cysteiny leukotreine 1 (CysLT1) and CysLT2 receptors.

Lipoxins (LXs) are generated from AA, EPA and DHA by transcellular biosynthetic mechanisms involving two cell populations. Neutrophils produce intermediates in LX synthesis, and these are converted to LXs by platelets interacting with leukocytes. LXA_4 _and LXB_4 _are generated by the action of platelet 12-lipoxygenase on neutrophil-derived LTA_4_. LXs inhibit leukocyte recruitment, neutrophil chemotaxis and adhesion to endothelium [7]. LXs have a negative regulation on LT synthesis and action and help in the resolution of inflammation. An inverse relationship generally exists between LXs and LTs and the balance between these two molecules appears to be crucial in the determination of degree of inflammation and its final resolution [4,6].

### Aspirin-triggered 15 epimer LXs (ATLs), resolvins and protectins

The formation of aspirin-triggered 15 epimer LXs (ATLs) are potent counter regulators of polymorphonuclear neutrophils (PMNs)-mediated injury and acute inflammation. Acetylated COX-2 enzyme of endothelial cells generates 15R-hydroxyeicosatetraenoic acid (15R-HETE) from AA that is converted by activated PMNs to the 15-epimeric LXs that have potent anti-inflammatory properties [2-9]. This cross-talk between endothelial cells and PMNs leading to the formation of 15R-HETE and its subsequent conversion to 15-epimeric LXs by aspirin-acetylated COX-2 is a protective mechanism to prevent local inflammation on the vessel wall by regulating the motility of PMNs, eosinophils, and monocytes [9]. Endothelial cells also oxidize AA, EPA and DHA via P450 enzyme system to form various hydroxyeicosatetraenoic acids and epoxyeicosatrienoic acids such as 11,12-epoxy-eicosatetraenoic acid(s) that have many biological actions that include blocking endothelial cell activation, while non-enzymatic oxidation products of EPA inhibit phagocyte-endothelium interaction and suppress the expression of adhesion molecules [10-15].

Akin to the formation of 15R-HETE and 15-epimeric LXs from AA, similar compounds are also formed from EPA and DHA. In the presence of aspirin, activated COX-2 of human endothelial cells converts EPA to 18R-HEPE, 18-HEPE, and 15R-HEPE. Activated human PMNs, in turn, converted 18R-HEPE to 5,12,18R-triHEPE and 15R-HEPE to 15-epi-LXA_5 _by their 5-lipoxygenase. Both 18R-HEPE and 5,12,18R-triHEPE inhibited LTB_4_-stimulated PMN transendothelial migration. 5,12,18R-triHEPE effectively competed with LTB_4 _for its receptors and inhibited PMN infiltration suggesting that it suppresses LT-mediated responses at the sites of inflammation [4,6,16,17].

The conversion of EPA by human endothelial cells with upregulated COX-2 treated with ASA of EPA to 15-epi-LX, also termed aspirin-triggered LX [ATL] and to 18R-hydroxyeicosapentaenoic acid (HEPE) and 15R-HEPE is interesting. These compounds in turn, are used by polymorphonuclear leukocytes to generate separate classes of novel trihydroxy-containing mediators, including 5-series 15R-LX(5) and 5,12,18R-triHEPE, which are potent inhibitors of human polymorphonuclear leukocyte transendothelial migration and infiltration in vivo (ATL analogue > 5,12,18R-triHEPE > 18R-HEPE). Acetaminophen and indomethacin also permitted 18R-HEPE and 15R-HEPE generation with recombinant COX-2. The formation of these bioactive lipid mediators via COX-2-nonsteroidal antiinflammatory drug-dependent oxygenations and cell-cell interactions may have significant therapeutic benefits in inflammation [16,17].

Murine brain cells expressing COX-2 and treated with aspirin transformed enzymatically DHA to 17R series of hydroxy DHAs (HDHAs) that, in turn, is converted enzymatically by PMNs to di- and tri-hydroxy containing docosanoids [16-19]. The conversion of DHA by leukocytes, brain, and glial cells to 17S-hydroxy-containing docosanoids denoted as docosatrienes (the main bioactive member of the series was 10,17S-docosatriene) and 17S series resolvins serve as regulators of both leukocytes reducing infiltration in vivo and glial cells blocking their cytokine production. These results indicate that DHA is the precursor to potent protective mediators generated via enzymatic oxygenations to novel docosatrienes and 17S series resolvins that have significant anti-inflammatory action and participate in the resolution of inflammatory events.

Similar small molecular weight compounds are also generated from AA, EPA, and DHA: 15R-hydroxy containing compounds from AA, 18R series from EPA, and 17R-hydroxy series from DHA. All these compounds have potent anti-inflammatory actions, resolve inflammation and hence are called as "resolvins". Resolvins inhibited cytokine generation, leukocyte recruitment, leukocyte diapedesis, and exudate formation. The formation of resolvins from AA, EPA, and DHA from acetylated COX-2 are generated via transcellular biosynthesis (e.g. due to cell-cell communication between endothelial cells and PMNs), and their main purpose appears to be to suppress inflammation. Resolvins inhibited brain ischemia-reperfusion injury [18,19]. It is possible that lipoxins, resolvins and protectins (docosanoids are also called as protectins since they have neuroprotective actions) behave as endogenous anti-inflammatory and cytoprotective molecules. The general cytoprotective properties that have been attributed to AA, EPA, and DHA can be related to their conversion to lipoxins, resolvins and docosanoids (protectins). Hence, any defect in the synthesis of lipoxins, resolvins and protectins or their inappropriate degradation could lead to perpetuation of inflammation as seen in lupus and other rheumatological conditions.

### Anti-inflammatory molecule lipoxin A_4 _is detectable in urine

LX_4_, generated by lipoxygenase (LO) transformation of AA possess potent anti-inflammatory activity in vivo, and temporal biosynthesis of LX, concurrent with spontaneous resolution, has been observed during exudate formation [8]. Recently, a new extraction technique, more selective for LX, which abolishes background contamination and minimizes the unspecific reading, was developed. This new method showed that urine from healthy subjects contains LXA_4 _[20]. Subsequently, it was shown [21] that strenuous exercise significantly increased urinary excretion in healthy volunteers (0.061 ± 0.023 vs. 0.113 ± 0.057 ng/mg creatinine; P = 0.028). These findings confirm that alterations in the urinary excretion of LXA_4 _can used as a reflection of changes in its (LXA_4_) formation, especially in the kidney, to monitor changes in the systemic and renal inflammatory lesions.

In a further extension of this work, it was reported that urinary levels of LXA_4 _was decreased while that of cysLTs (cysteinyl leukotrienes) increased in volunteers aged from 26 to over 100 years leading to a profound unbalance of the LXA(4)/cysLTs ratio that may be considered an index of the endogenous anti-inflammatory potential [22]. These results suggest that endogenous anti-inflammatory mechanisms become less efficient with age that could result in increased susceptibility to inflammatory disorders with advancing age.

### Urinary LXA_4 _is decreased and LTs increased in HSP nephritis

In a study in which temporal changes of blood and urinary LXA_4_, LTB_4 _(leukotriene B_4_) and urinary LTE_4 _was determined in 49 children with Henoch-Schonlein purpura (HSP) in which renal inflammation is known to occur, it was reported that inverse temporal changes between gradually increased blood and urinary LXA_4 _and gradually decreased blood and urinary LTB_4 _and urinary LTE_4_. Furthermore, both 15-S-hydroxyeicosatetraenoic acid and LXA_4 _inhibited the LTB_4_-induced chemotaxis of leukocytes and release of LTB_4 _from leukocytes obtained from the patients in the active phase of HSP. In 22 children with HSP nephritis, concordant with the gradually increased severity of mesangial proliferation and proteinuria, the glomerular expressions of 15-lipoxygenase and the levels of urinary LXA_4 _gradually decreased and the glomerular expressions of LTC_4 _synthase and the urinary LTE_4 _and LTB_4 _gradually increased. The levels of blood and urinary LXA_4 _in patients with HSP nephritis were lower than those in patients with purpura alone in early resolution of HSP. The levels of blood and urinary LTB_4 _and urinary LTE_4 _in the patients with HSP nephritis were higher than those in patients with purpura alone in early resolution of HSP. A positive correlation between blood LTB_4 _and serum C-reactive protein in 49 children with HSP was also noted [23]. These data suggest that LTs play a proinflammatory and profibrotic role in the pathogenesis of HSP, and insufficiency of LXA_4 _may be responsible for the patients with HSP whose illness become more serious. These results suggest that absence or decreased levels of LXA_4 _that acts as a "stop signal" of inflammatory process may be responsible for the nephritis seen in HSP.

### Anti-inflammatory cytokines IL-4 and IL-10 enhance LXA_4 _synthesis

In this context, it is noteworthy that anti-inflammatory cytokines IL-4 and IL-10 trigger the conversion of AA, EPA and DHA to lipoxins, resolvins, protectins and maresins suggesting a mechanism by which they are able to suppress inflammation [24]. For example, IL-4 up-regulated {(it may be noted here that 15-S-HETE (a 15-LO product; LO = lipoxygenase enzyme) and lipoxins (interaction products between 5-LO and either 12-LO or 15-LO) counteract the proinflammatory actions of leukotrienes} 15-LO gene expression in human leukocytes. Glomerular 12/15-LO mRNA increased significantly over controls 24 and 48 hours after nephrotoxic serum injection then decreased at 72 hours. RNA from nephrotoxic serum injected glomeruli contained higher levels of 12/15-LO mRNA than that from unstimulated peripheral leukocytes, suggesting that 12/15-LO transcription is up-regulated locally in native and/or infiltrating glomerular cells. Glomerular IL-4 mRNA increased markedly 16 hours post-nephrotoxic serum injection, and was then reduced, suggesting a potential role for T cell-derived IL-4 in directing the expression of 12/15-LO during glomerulonephritis. This suggested tandem regulated in vivo gene expression for IL-4 and LO, both of which promote counter-inflammatory influences in immune complex-mediated injury.

### Balance between LXA_4 _and LTs determines the degree of systemic and glomerular inflammation

The 5-lipoxygenated metabolites of AA, the leukotrienes, are major mediators of early glomerular hemodynamic and structural deterioration during experimental glomerulonephritis that are generated largely by infiltrating leukocytes, but can also occur by intrinsic glomerular cells via transcellular metabolism of intermediates. In animal models of glomerulonephritis and other renal pathologic states, leukotrienes have been shown to exert adverse effects in the glomerulus. LTB_4 _augments neutrophil infiltration, and LTC_4 _and LTD_4 _mediate potent vasoconstrictor effects on the glomerular microcirculation. Selective blockade of the 5-lipoxygenase pathway produced a significant amelioration of the deterioration of renal hemodynamic and structural parameters. On the other hand, 15-S-hydroxyeicosatetraenoic acid (15-S-HETE), the immediate product of arachidonate 15-lipoxygenase, and the lipoxins, which are produced by sequential 15- and 5- or 5- and 12-lipoxygenation of AA are also generated in the course of glomerular injury that antagonize leukotriene-induced neutrophil chemotaxis and lipoxin A_4 _antagonized the effects of LTD_4 _and LTC_4 _on the glomerular microcirculation. Thus, the contrasting effects of 5- and 15-lipoxygenase products represent endogenous pro- and anti-inflammatory influences that ultimately determine and regulate the extent and severity of glomerular inflammation [25-28].

These results are in favor of the proposal that anti-inflammatory cytokines IL-4 and IL-10 induce the expression and synthesis of anti-inflammatory lipid mediators lipoxins, resolvins, protectins and maresins in addition to their ability to suppress the production of pro-inflammatory cytokines such as IL-2, IL-6, TNF-α, MIF and HMGB1 and LTs.

### PUFAs and lipoxins bind to GPCR to suppress inflammation

It is noteworthy that monocytes and macrophages express an extensive repertoire of G protein-coupled receptors (GPCRs) that regulate inflammation and immunity. A number of GPCRs (e.g. *Edg5*, *P2ry2 *and *6*) have been reported to be expressed by macrophages, and two cell types closely related to macrophages (osteoclasts and dendritic cells), whereas *Gpr84 *expression was largely restricted to macrophage populations and granulocytes [29]. It is now apparent that many PUFAs, especially AA, EPA and DHA and their metabolites such as eicosanoids, lipoxins, resolvins, protectins and maresins also function directly as agonists at a number of G protein-coupled receptors (GPCRs). Tissue distribution studies and siRNA knock-down experiments have indicated key roles for these GPCRs in glucose homeostasis, adipogenesis, leukocyte recruitment and inflammation [30]. A recent study showed that the G protein-coupled receptor 120 (GPR120) functions as a ω-3 fatty acid receptor/sensor. Stimulation of GPR120 with ω-3 fatty acids (EPA and DHA) induced broad anti-inflammatory effects in monocytic RAW 264.7 cells and in primary intraperitoneal macrophages. All of these effects were abrogated by GPR120 knockdown. The ω-3 fatty acid treatment not only inhibited inflammation but also enhanced systemic insulin sensitivity in wild-type mice, but was without effect in GPR120 knockout mice. These results suggest that GPR120 is a functional ω-3 fatty acid receptor/sensor and mediates potent insulin sensitizing and antidiabetic effects in vivo by repressing macrophage-induced tissue inflammation [31]. Thus, it is likely that PUFAs and their anti-inflammatory products such as lipoxins, resolvins, protectins and maresins inhibit the production of various pro-inflammatory molecules including MIF and HMGB1 and thus, suppress inflammation in diseases such as lupus and rheumatoid arthritis.

### Hypothesis

#### A deficiency of LXA_4 _and excess of LTs may be responsible for lupus/lupus nephritis

Based on the evidences presented above and the role of LXA_4 _and LTs in inflammation, it is quite logical to suggest that a deficiency of LXA_4 _and/or an excess of LTs initiate and perpetuate systemic and renal inflammation in lupus. Since, it is possible to estimate these compounds in the urine; I propose that progression and flares of lupus and lupus nephritis are due to decreased production of LXA_4 _and enhanced production of LTs by the renal tissue and/or infiltrating leukocytes and macrophages. These molecules can be detected and estimated in the urine [20-23] as already discussed above. Furthermore, the urinary levels of LXA_4 _and LTs may also be used to predict prognosis and response to treatment. If the urinary LXA_4 _levels revert to normal or are slowly increasing with and without decrease in urinary levels of LTs, it can be considered as an indication that the patient is responding to treatment and that both systemic and renal lesions of lupus are ameliorating. The urinary levels of LXA_4 _and LTs can be compared to their plasma concentrations; and to serum urea, creatinine and urinary protein values and wherever possible to renal biopsy findings to know the progress of renal lupus disease.

Since resolvins, protectins and maresins are also anti-inflammatory lipid molecules derived from EPA and DHA and have a role in the resolution of inflammation, it is predicted that they may also have the same significance as that of lipoxins in lupus nephritis. To measure resolvins, protectins and maresins in urine reliable methods are yet to be developed. If they are developed, then it is worthwhile to measure the urinary levels of resolvins, protectins and maresins in addition to lipoxins in the urine of patients with lupus and lupus nephritis to predict prognosis and response to treatment. Decrease in their levels in the urine indicates continued inflammation and renal damage whereas enhancement in their levels indicates resolution of inflammation and amelioration of lupus nephritis.

### Therapeutic implications

Based on this hypothesis, it is proposed that LXA_4 _and its more stable synthetic analogues and resolvins, protectins, maresins and their agonists are useful in the management of lupus, rheumatoid arthritis, type 1 diabetes mellitus and other autoimmune diseases. Measurement of plasma and urinary levels of LXA_4_, resolvins, protectins and maresins may be used as prognostic markers in these diseases, while in multiple sclerosis; measurement of cerebrospinal fluid (CSF) levels of LXA_4_, resolvins, protectins and maresins can be used as markers of disease progression, response to therapy and its prognosis.

**Figure 1:**
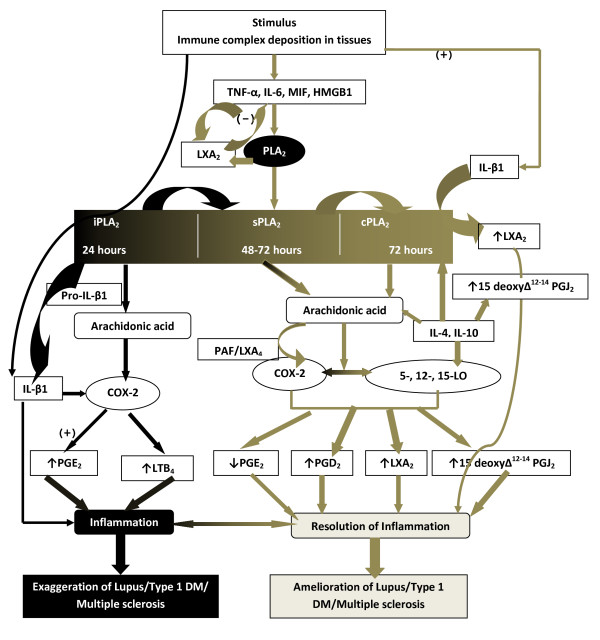
**Scheme showing the role of prostaglandins, leukotrienes and lipoxins in lupus and other autoimmune diseases**. (-) Indicates inhibition or suppression of action; (+) Indicates activation or enhancement of action. There are three classes of phospholipases that control the release of AA and other PUFAs: calcium-independent PLA_2 _(iPLA_2_), secretory PLA_2 _(sPLA_2_), and cytosolic PLA_2 _(cPLA_2_) [40]. Each class of PLA_2 _is further divided into isoenzymes for which there are 10 for mammalian sPLA_2_, at least 3 for cPLA_2_, and 2 for iPLA_2_. During the early phase of inflammation, COX-derived PGs and lipoxygenase-derived LTs initiate exudate formation and inflammatory cell influx [41]. TNF-α causes an immediate influx of neutrophils concomitant with PGE_2 _and LTB_4 _production, whereas during the phase of resolution of inflammation an increase in LXA_4 _(lipoxin A_4_), PGD_2 _and its product 15deoxyΔ^12-14^PGJ_2 _formation occurs that induces resolution of inflammation with a simultaneous decrease in PGE_2 _synthesis that stops neutrophil influx and enhances phagocytosis of debris [42,43]. Thus, there appears to be two waves of release of AA and other PUFAs: one at the onset of inflammation that causes the synthesis and release of PGE_2 _and a second at resolution for the synthesis of anti-inflammatory PGD_2_, 15deoxyΔ^12-14^PGJ_2_, and lipoxins that are necessary for the suppression of inflammation. Thus, COX-2 enzyme has both harmful and useful actions by virtue of its ability to give rise to pro-inflammatory and anti-inflammatory PGs and LXs. Increased type VI iPLA_2 _protein expression was found to be the principal isoform expressed from the onset of inflammation up to 24 hours, whereas type IIa and V sPLA_2 _was expressed from the beginning of 48 hours till 72 hours while type IV cPLA_2 _was not detectable during the early phase of acute inflammation but increased progressively during resolution peaking at 72 hours. This increase in type IV cPLA_2 _was mirrored by a parallel increase in COX-2 expression [44]^5^. The increase in cPLA_2 _and COX-2 occurred in parallel, suggesting a close enzymatic coupling between these two. Thus, there is a clear-cut role for different types of PLA_2 _in distinct and different phases of inflammation. Selective inhibition of cPLA_2 _resulted in the reduction of pro-inflammatory molecules PGE_2_, LTB_4_, IL-1β, and platelet-activating factor (PAF). Furthermore, inhibition of types IIa and V sPLA_2 _not only decreased PAF and LXA_4 _(lipoxin A_4_) but also resulted in a reduction in cPLA_2 _and COX-2 activities. These results suggest that sPLA_2_-derived PAF and LXA_4 _induce COX-2 and type IV cPLA_2_. IL-1β induced cPLA_2 _expression. This suggests that one of the functions of IL-1 is not only to induce inflammation but also to induce cPLA_2 _expression to initiate resolution of inflammation [45,46]. Synthetic glucocorticoid dexamethasone inhibited both cPLA_2 _and sPLA_2 _expression, whereas type IV iPLA_2 _expression is refractory to its suppressive actions [44,47,48]. Activated iPLA_2 _contributes to the conversion of inactive pro-IL-1β to active IL-1β, which in turn induces cPLA_2 _expression that is necessary for resolution of inflammation. LXs, especially LXA_4 _inhibit TNF-α-induced production of ILs; promote TNF-α mRNA decay, TNF-α secretion, and leukocyte trafficking and thus attenuates inflammation.

Previously, I showed that AA, EPA and DHA prevented chemical-induced type 1 DM in experimental animals [32-34]. Recent studies [35-39] both in experimental animals and humans confirmed that PUFAs, especially n-3 fatty acids, do prevent type 1 DM. Since AA, EPA and DHA form precursors to LXs, resolvins, protectins and maresins, the ability of these fatty acids to prevent DM can be ascribed to the formation of the anti-inflammatory lipids [37-39]. Thus, PUFAs and LXs, resolvins, protectins and maresins are expected to be beneficial in lupus, RA, multiple sclerosis and other autoimmune diseases (see Figure 1).

## Conflict of interest statement

The authors declare that they have no competing interests.
